# Mistletoe lectin inhibits growth of Myc‐amplified small‐cell lung cancer

**DOI:** 10.1002/cam4.5558

**Published:** 2022-12-23

**Authors:** Mohammad A. Shatat, Betsy Gauthier, Suzy Yoon, Eric Yuan, Peiying Yang, Goutham Narla, Afshin Dowlati, Richard T. Lee

**Affiliations:** ^1^ Division of Pulmonary, Critical Care and Sleep Medicine Case Western Reserve University The Louis Stokes Cleveland VA Medical Center and Case Comprehensive Cancer Center Ohio Cleveland USA; ^2^ Case Comprehensive Cancer Center Case Western Reserve University Ohio Cleveland USA; ^3^ Departments of Palliative, Rehabilitation, and Integrative Medicine The University of Texas MD Anderson Cancer Center Texas Houston USA; ^4^ Division of Genetic Medicine Department of Internal Medicine The University of Michigan Michigan Ann Arbor USA; ^5^ Departments of Supportive Care Medicine and Medical Oncology City of Hope Comprehensive Cancer Center California Duarte USA

**Keywords:** mistletoe, MYC, natural products, small‐cell lung cancer, targeted therapy

## Abstract

**Background:**

Small‐cell lung cancer (SCLC) is the deadliest form of lung cancer but lacks targeted therapies.

**Methods:**

We studied the effect of the natural product mistletoe lectin (ML) in pre‐clinical models of SCLC, focusing on cell lines with amplification of the myc family oncogenes C‐myc and N‐myc.

**Results:**

We found that ML treatment inhibits growth of SCLC cell lines in culture and induces apoptosis. ML treatment also decreases the expression of the amplified myc proteins. Over‐expression of either C‐myc or N‐myc results in enhanced SCLC cell sensitivity to ML. In a mouse xenograft model of SCLC, treatment with ML results in decreased tumor growth over 4 weeks with evidence of increased apoptosis in tumors from treated animals.

**Conclusion:**

Overall, our results demonstrate that ML exhibits therapeutic potential in SCLC, that is at least partially dependent on myc protein expression.

## 1 INTRODUCTION

Small‐cell lung cancer (SCLC) accounts for approximately 15% of all lung cancers.^1^ Platinum‐based chemotherapy remains the mainstay of treatment,^2^ with the recent approval of the addition of immune checkpoint inhibitors based on survival benefit in extensive‐stage disease.^3^, ^4^ But even with treatment, prognosis remains poor, with a median survival of up to 13 months in patients with extensive stage disease. A targeted therapy directed at the credentialed molecular drivers of the disease remains lacking.

Approximately 20% of untreated SCLC tumors have amplification of one of the myc family proto‐oncogenes: C‐myc (also known as MYC), N‐myc, and L‐myc.^5^, ^6^, ^7^ MYC pathway activation plays an important role in tumorigenesis: Expression of any one of these three myc genes accelerates tumor growth in genetically engineered mouse models of SCLC.^8^, ^9^, ^10^ N‐myc has also been demonstrated to be a driver of chemoresistance in SCLC.^9^ Moreover, the amplification of C‐myc and N‐myc in clinical SCLC samples has been associated with a worse prognosis.^11^ These findings present a rationale for exploring myc family proto‐oncogenes as therapeutic targets in SCLC.

European Mistletoe (*Viscum album*) refers to a hemiparasitic plant, part of the order Santalales. Extracts of the plant mistletoe have been shown to have anti‐cancer effects in pre‐clinical studies.^12^ Mistletoe lectin (ML) is a heteroprotein complex composed of A‐ and B‐chains connected by a disulfide bond. The A‐chain has been reported to inhibit ribosomes, while the B‐chain binds to cell surface glycoproteins triggering the internalization of ML.^13^ We have previously shown that ML inhibits the growth of hepatocellular carcinoma, in part by down‐regulating C‐myc expression.^14^

Here, we report the effect of treatment with ML on SCLC cell lines in culture and in a mouse xenograft model of the disease. In light of our prior finding of C‐myc down‐regulation in hepatocellular carcinoma, we sought to explore whether the effect of ML treatment in SCLC is at least partially mediated through modulation of myc expression. We also examined the effect of ML on the expression of C‐myc and N‐myc, since aberrant expression of both proteins has been associated with accelerated tumor growth and poor prognosis in SCLC. This represents a novel approach by utilizing a natural product for targeted therapy in this subset of SCLC.

## 2 MATERIALS AND METHODS

### 2.1 Cell culture

Human SCLC cell lines were cultured at 37°C and 5% CO_2_. H82, H69, H196, and SHP77 cells were obtained from the American Type Culture Collection (ATCC) and maintained in Hyclone RPMI 1640 medium (Cytvia) with 10% fetal bovine serum (vWR), with or without antibiotics (50 units of penicillin and 50 μg of streptomycin, Cytvia). If cells were cultured for more than 3 years, they were validated by STR profiling at the ATCC.

### 2.2 Chemicals

Mistletoe lectin was from Sigma. Cisplatin was from Cayman Chemical.

### 2.3 Animals

All animal experiments were approved by the IACUC at Case Western Reserve University. Athymic nude mice (NU/J) were purchased from the Jackson Labs and maintained under isolation conditions at the athymic core facility of Case Western Reserve University.

### 2.4 Tumor xenografts

H82 cells (4 × 10^6^/mouse) in serum‐free media were mixed 1:1 with Matrigel® and injected into the right flank of athymic mice. The resulting tumors were measured with a caliper, and volume was calculated using the formula (length × width^2^/2). When tumor volumes reached an average of 100 mm^3^, the mice were randomized to four treatment conditions: PBS, ML, cisplatin, and ML + cisplatin. ML was administered as a subcutaneous injection at a dose of 2 μg/kg three times per week. This dose was chosen because in pilot mouse experiments we found it to be well‐tolerated and there was no additional inhibition of tumor growth when the dose was increased further (Figure S3). Cisplatin was administered as an intraperitoneal injection at a dose of 4 mg/kg weekly.^15^

### 2.5 Immunohistochemistry

Formalin‐fixed and paraffin‐embedded tumor xenograft samples were sectioned at 5‐micron thickness. Slides were deparaffinized by incubating in Xylene twice, followed by graded ethanol incubation and a rinse with de‐ionized water. They were then double‐stained with an antibody cocktail for caspase‐3 and Ki‐67 purchased from Biocare Medical (Cat. no. PPM 240 DS AA) following the manufacturer's instructions. Whole stained slides were then scanned using Hamamatsu Nanozoomer S60, and quantification of caspase‐3 and Ki‐67 was performed with ImageJ software (NIH).

### 2.6 Antibodies

C‐myc and PARP primary antibodies were purchased from Cell Signaling (Cat. no. 9402 and 9542, respectively). N‐myc, vinculin, and GAPDH primary antibodies were purchased from Abcam (Waltham, MA, USA. Cat no. ab16898) and Santa Cruz Biotechnology (Cat. no. sc‐73614 and sc‐32233, respectively). All Fluorescence secondary antibodies were purchased from Li‐COR Biosciences (Cat no. 925‐32213, 925‐32212, and 925‐68070).

### 2.7 Immunoblotting

Cells were harvested with RIPA buffer (Thermo Fisher Scientific) containing protease inhibitors (Roche). Total protein was separated on a 4–20% gradient SDS gel (Bio‐rad) and was transferred to a nitrocellulose membrane(Bio‐rad). The membranes were blocked with 5% non‐fat milk in Tris‐buffered saline solution and probed with primary antibodies overnight at 4°C. To ensure equal loading and transfer, membrane total protein staining was performed using Revert™700 stain (Li‐COR Biosciences) following the manufacturer's instructions. Immunoblot image acquisition and analysis were performed with Image Studio software (Li‐COR Biosciences).

### 2.8 Cell viability analysis

Cells were cultured in 96‐well plates and treated with increasing concentrations of ML for 48 h. Viability was determined with a colorimetric MTS assay using CellTiter 96® AQueous Non‐Radioactive Cell Proliferation Assay (Promega). To analyze the effect of combining ML and cisplatin, cells were treated with different concentrations of the two drugs, and viability was determined using the CellTiter‐Glo® Luminescent Cell Viability Assay (Promega). Results were analyzed using SynergyFinder Plus web application.^16^

### 2.9 Apoptosis analysis

Cells were treated with the indicated concentrations of ML for 24 or 48 h, then stained for Annexin V and Propidium iodide (PI) using Alexa Fluor® 488 annexin V/Dead Cell Apoptosis Kit from Invitrogen by Thermo Fisher Scientific (Cat. no. V13241) following the manufacturer's instructions. Fluorescence was detected with an Attune™ NxT Flow Cytometer (Thermo Fisher Scientific), and results were analyzed with FlowJo™ software.

### 2.10 Enzyme activity

Calpain activity was measured using the calpain assay kit from Abcam (cat# ab65308) and following the manufacturer's instructions. Briefly, cells were treated with different concentrations of ML in a 6‐well plate and following cell lysis, equal amounts of protein were incubated with the calpain substrate in a 96‐well plate and a fluorometric assay was used to measure calpain activity. Caspase 3 and caspase 7 activities were measured using Caspase‐Glo® 3/7 Assay System from Promega (cat# G8091). Briefly, cells were treated with different concentrations of ML in 96‐well plates and the caspase 3/7 substrate was added to each well. A luminometric assay was used to measure caspase 3/7 activity. Promega GloMax® Explorer microplate reader was used in both assays.

### 2.11 Lentivirus transduction

pLX307 hcRed1 was a gift from William Hahn (Addgene plasmid # 117732). MYC_pLX307 was a gift from William Hahn & Sefi Rosenbluh (Addgene plasmid # 98363). Plasmids for N‐myc and control vector with GFP were obtained from GeneCopoeia (Cat. no. EX‐Y2047‐Lv102 and EX‐EGFP‐Lv102, respectively). Each plasmid was co‐transfected with pMD2.G and psPAX2 helper plasmids into HEK293T cells using Lipofectamine™ 3000 Transfection Reagent (Invitrogen) and following the manufacturer's protocol. After 48 h, lentiviruses were collected from the supernatant to infect SHP77 cells in the presence of 8 μg/ml polybrene (Santa Cruz). The growth medium was replenished with medium containing 2 μg/ml puromycin (Thermo Fisher Scientific) 48 h after infection for transduced cell clone selection.

### 2.12 Quantitative PCR

Total RNA was extracted using NucleoSpin® RNA kit (Takara Bio). cDNA was synthesized using qScript cDNA Synthesis Kit (Quanta Bio). Quantitative PCR was performed using Applied Biosystems™ Power SYBR™ Green PCR Master Mix (Thermo Fisher Scientific) on a LightCycler® 480 System (Roche). Primer sequences are listed in Table S1.

### 2.13 Data analysis

All cell culture experiments were performed in three independent replicates. Data were expressed as means ± SD. Student's t‐test was used for two‐group comparisons and ANOVA with post hoc Tukey analysis was used for multiple group comparisons. *p* values less than 0.05 were considered statistically significant. Data were analyzed using Graphpad Prism software.

## 3 RESULTS

### 3.1 ML inhibits growth in SCLC cell lines

**FIGURE 1:**
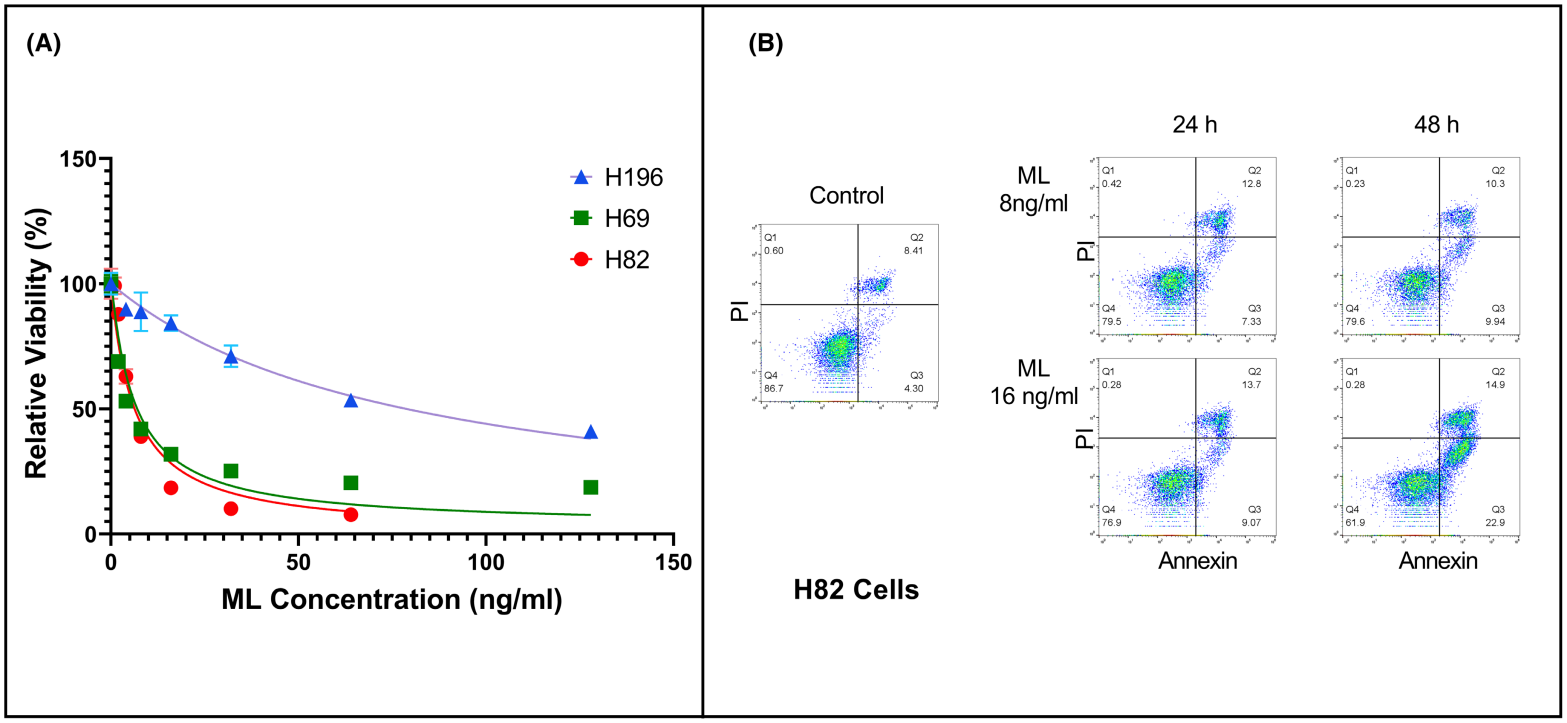
ML inhibits growth and induces apoptosis in human SCLC lines. (A) Viability of three SCLC cell lines after being treated for 48 h with increasing concentrations of ML. Viability was assessed with the MTS assay (CellTiter 96® Aqueous) and is expressed as absorbance of treated cells relative to untreated cells. (B) Flow cytometry analysis of H82 cells treated with ML. Annexin V is represented on the X axis and propidium iodide (PI) on the Y axis. Cells undergoing apoptosis with only Annexin V staining are in quadrant 3 (lower right).

We tested the effect of ML treatment on three SCLC cell lines: NCI‐H82, which has C‐myc amplification, NCI‐H69, which has N‐myc amplification, and NCI‐H196, which does not have amplification of any myc family genes and does not express significant amounts of myc proteins. Following treatment with increasing ML concentrations for 48 h, we analyzed cell viability with the MTS assay and calculated the GI50 for each cell line. We found that H82 and H69 cells had similar responses to ML treatment with GI50 of 6.3 and 6.2 ng/ml, respectively (no significant difference between the two, *p* = 0.91), but H196 cells were less sensitive to ML treatment, with GI50 of 78.7 ng/ml (*p* < 0.0001 when comparing GI50 for H196 with that of either H82 or H69) (Figure 1A and Table S2).

### 3.2 ML treatment induces apoptosis in H82 cells

To assess whether the decrease in cell viability is in part due to the induction of apoptosis, we treated H82 cells with two different concentrations of ML and analyzed for apoptosis at 24 and 48 h using Annexin V staining and flow cytometry. We found that ML treatment induced apoptosis in a concentration‐dependent and time‐dependent manner (Figure 1B and Table S3).

### 3.3 ML treatment decreases protein expression of C‐myc in H82 cells and N‐myc in H69 cells and increases PARP cleavage in both cell lines

**FIGURE 2:**
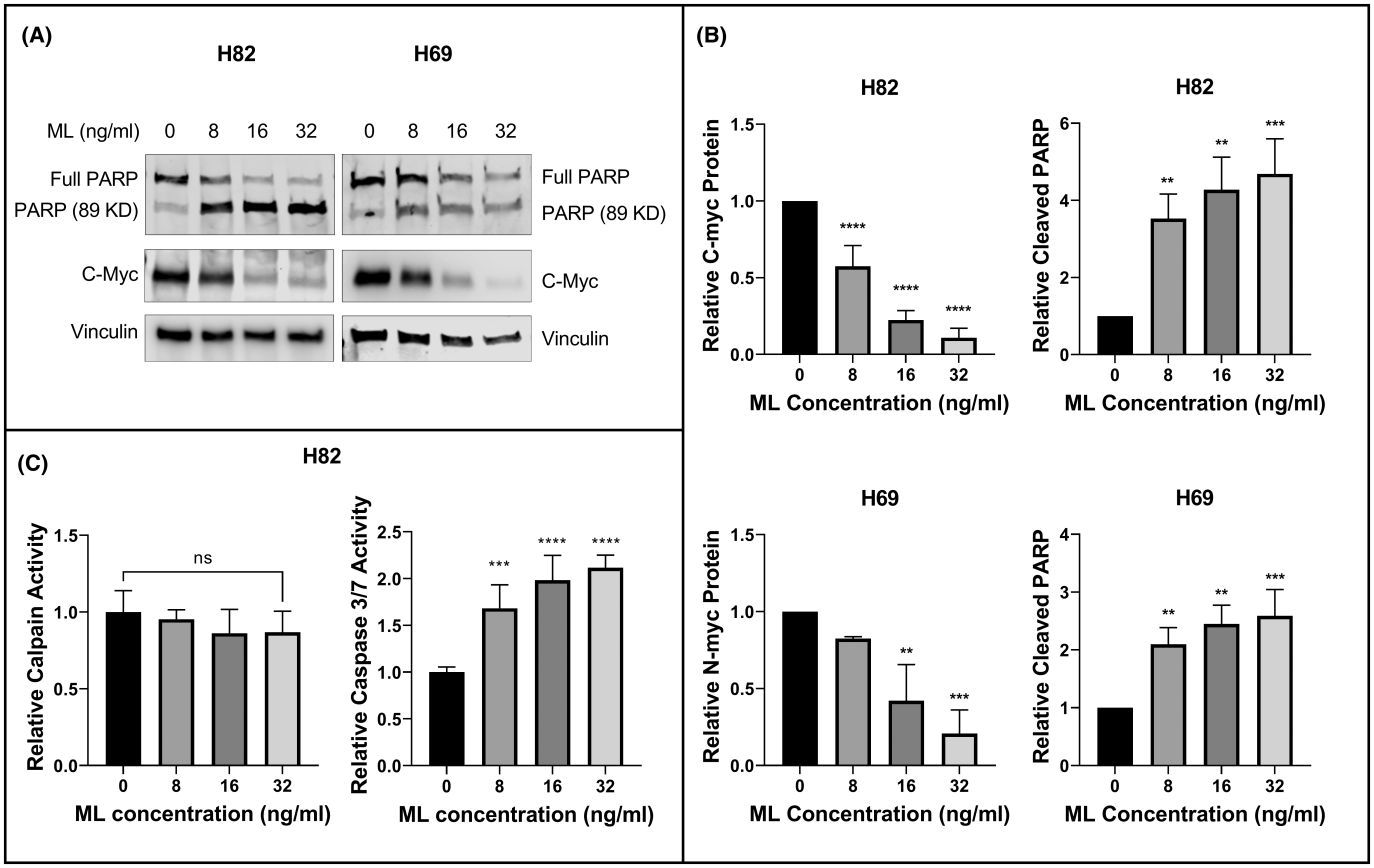
Mistletoe lectin (ML) decreases expression of myc proteins and induces PARP cleavage in SCLC lines. (A) Immunoblot of H82 cells with C‐Myc and PARP and H69 cells with N‐Myc and PARP following incubation with the indicated concentrations of ML for 24 h. Vinculin was used as loading control. (B) Graphic representation of protein expression shown in panel A, calculated using three independent experiments. Relative C‐myc and N‐myc protein expression was normalized to loading control. Relative cleaved PARP was normalized to total PARP. (C) Enzyme activity of for calpain (left) and caspase 3/7 (right) in H82 cells. Calpain activity was measured with a fluorometric assay and caspase activity was measured with a luminometric assay that measures the activity of both caspase 3 and caspase 7. Results are expressed as a fold change relative to untreated H82 cells. **p < 0.01, ***p < 0.001, ****p < 0.0001 for comparison with the control group.

We next assessed the effect of ML treatment on myc protein expression in two SCLC cell lines: NCI‐H82, which has C‐myc amplification, and NCI‐H69, which has N‐myc amplification. We treated cultured cells with increasing concentrations of ML for 24 h. Western blotting showed a concentration‐dependent reduction in the respective myc protein expression with ML treatment. There was also an increase in PARP cleavage, a marker of apoptosis (Figure 2A,B). Densitometry analysis showed that with ML concentrations of 32 ng/ml in H82 cells, C‐myc level dropped by 95% compared to baseline (*p* < 0.0001), and cleaved PARP increased by 4.7 fold (*p* < 0.001). In H69 cells treated with ML concentrations of 32 ng/ml, N‐myc expression was decreased by 79% (*p* < 0.001), and cleaved PARP increased by 2.6 fold (*p* < 0.001). Densitometry analysis results are shown in Figure 2B.

To assess myc gene transcription, we treated H82 and H69 cells with ML for 24 h, extracted total RNA for RT‐QPCR analysis of C‐myc and N‐myc respectively. There was no drop in mRNA expression of either gene, but rather a trend of higher mRNA expression (Figure S2). This indicates that the drop in protein expression of C‐myc and N‐myc induced by ML is not mediated by decreased transcription.

### 3.4 ML Treatment induces activity of Caspase 3/7 but not calpain in H82 cells

To assess the mechanism by which PARP is cleaved following treatment of H82 cells with ML, we measured activity of calpain and the caspases 3 and 7, which are known to mediate PARP cleavage.^17^ Enzyme activities for caspase 3 and caspase 7 are measured by a single assay, denoted caspase 3/7. ML treatment of H82 cells for 24 h did not result in a significant change in calpain activity (Figure 2C, left panel). However, ML treatment induced caspase 3/7 activity by 2.1 fold (±0.13) (Figure 2C, right panel).

### 3.5 Over‐expression of C‐myc and N‐myc enhances the effect of ML on survival of SHP77 cells

**FIGURE 3:**
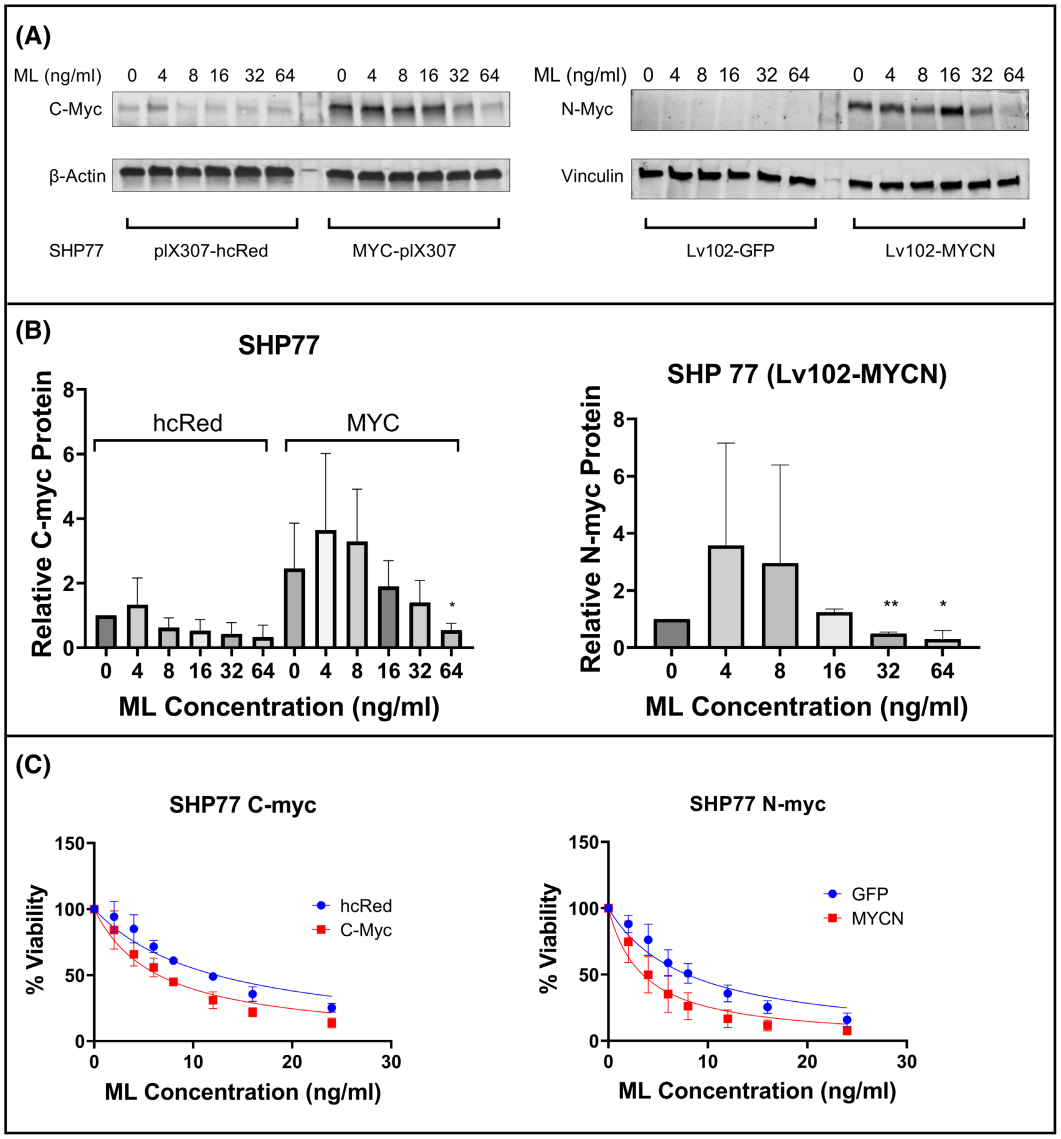
Forced expression of myc sensitizes SCLC lines to ML. (A) Immunoblot of transduced SHP77 cells with C‐Myc and N‐myc following incubation with indicated concentrations of ML. (B) Graphic representation of C‐myc and N‐myc protein expression shown in panel A, calculated from three independent experiments. Relative C‐myc and N‐myc protein expression were normalized to loading control. SHP77 cells transduced with Lv102‐GFP do not have detectable N‐myc expression and are therefore not represented on the graph. *p < 0.05, **p < 0.01 for comparison with PBS group. (C) Cell viability of transduced SHP77 with the MTS assay following a 48‐h incubation with increasing concentrations of ML.

To examine whether the effect of ML on SCLC cell lines is mediated by myc proteins, we utilized the SHP77 cell line, which does not have amplification of any of the myc family genes, but expresses detectable levels of C‐myc but not N‐myc. We over‐expressed C‐myc or N‐myc in these cells using lentiviral vectors. ML treatment resulted in decreased levels of the exogenously expressed myc proteins (Figure 3A,B). We found that over‐expression of C‐Myc resulted in increased sensitivity to ML treatment (GI50 12.3 with control vector and 6.5 with C‐myc over‐expression. *p* < 0.0001). Similarly, N‐myc over‐expression also increased sensitivity to ML treatment (GI50 7.9 with control vector and 3.4 with N‐myc over‐expression; *p* < 0.0001) (Figure 3C and Table S4).

### 3.6 ML treatment slows tumor growth in H82 mouse xenograft model

**FIGURE 4:**
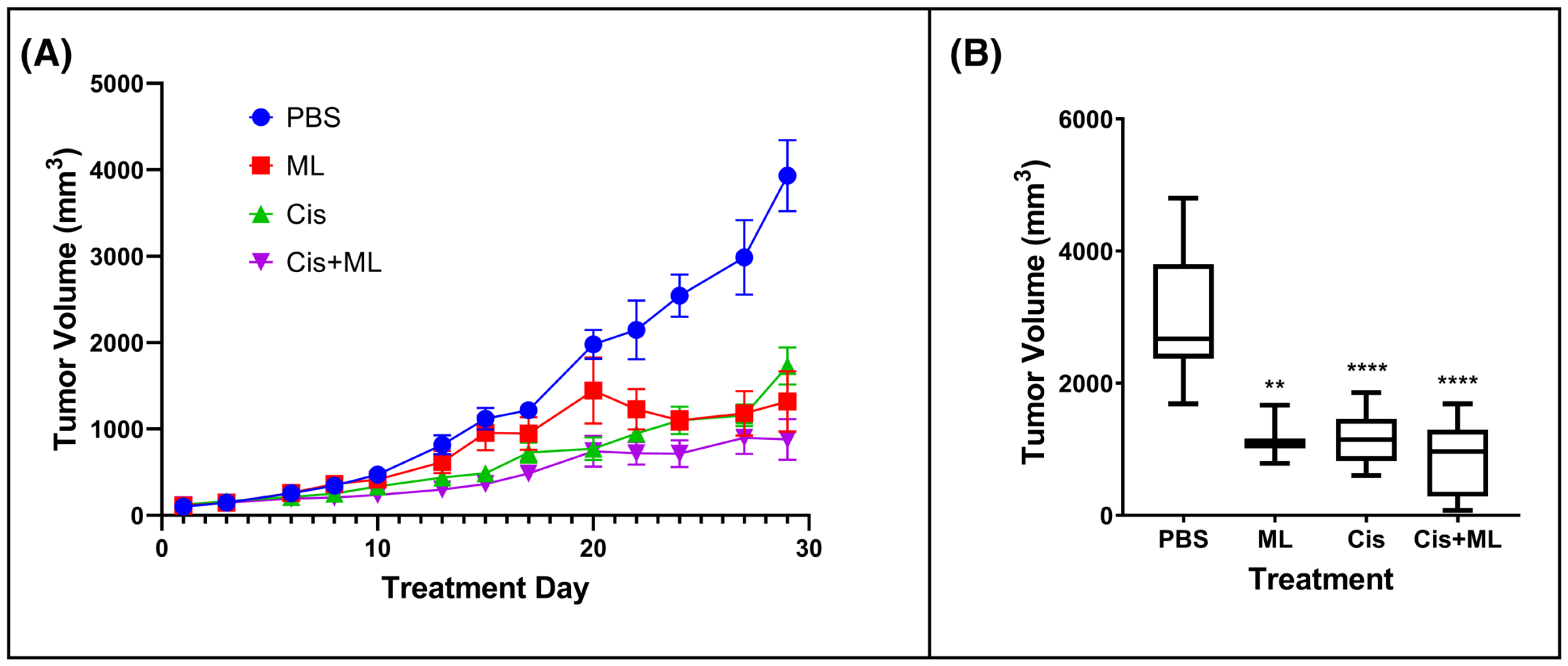
ML inhibits H82 xenograft tumor growth. (A) Tumor volume measurement over the course of treatment with: PBS (control), ML, cisplatin, or ML + cisplatin. (B) Tumor volume on day 27 of treatment. **p < 0.01, ****p < 0.0001 for comparison with PBS group.

To evaluate the efficacy of ML treatment in vivo, we utilized a mouse xenograft model derived from H82 cells in athymic nude mice. We treated the animals with either PBS as control, ML, cisplatin, or a combination of ML and cisplatin. We found that treatment with ML resulted in decreased tumor growth compared to PBS control (Figure 4A). After 4 weeks of treatment, all treatment groups had smaller tumor volumes than the control group, but there was no significant difference in tumor volumes between the ML group, the cisplatin group, and the combination group. On day 27, the mean tumor volume was 2984 mm^3^ for the PBS group, 1181 mm^3^ for the ML group, 1158 mm^3^ for the cisplatin group, and 899 mm^3^ for the cisplatin + ML group (Figure 4B). Comparison of excised tumor weights at the end of the 4‐week experiment showed a similar trend (Figure S4).

### 3.7 ML induces apoptosis in tumor xenografts and decreases proliferation when combined with cisplatin

**FIGURE 5:**
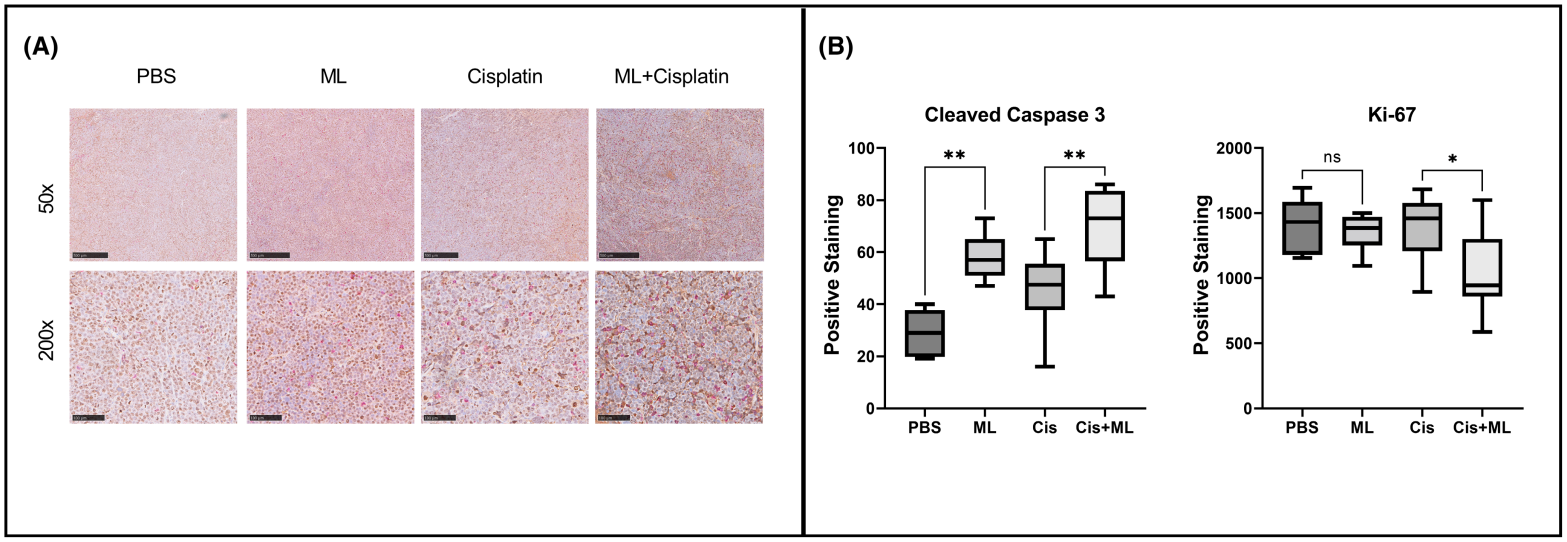
ML induces apoptosis in tumor xenografts and decreases cell proliferation when combined with cisplatin. (A) Representative photomicrographs of tumor xenograft sections from each treatment group stained for Ki‐67 (brown) and cleaved caspase 3 (red) show at 50× and 200× magnifications. Scale bars are 500 μm (top) and 100 μm (bottom). (B) Quantification of Ki‐67 staining (automated arbitrary units) and cleaved caspase staining (number of positive nuclei per HPF). *p < 0.05, **p < 0.01.

To further assess the effect of ML on H82 tumor xenografts, we stained sections of the tumors for Ki‐67, a marker for cell proliferation, and cleaved caspase 3, a marker of apoptosis (Figure 5A). Tumors treated with ML had an increased number of cells positive for cleaved caspase 3, indicating increased apoptosis. Cleaved caspase 3 positive cells per HPF were as follows: PBS 29.0 versus ML 57.8 (*p* = 0.006) and Cis 46.5 versus Cis + ML 69.8 (*p* = 0.0034). We found no significant difference in Ki‐67 staining quantity between the control and ML groups (1419 vs. 1356 arbitrary units (AU) respectively, *p* = 0.97). However, the addition of ML to cisplatin decreased Ki‐67 staining from 1389 AU for cisplatin alone to 1039 for cisplatin + ML (*p* = 0.029), indicating a reduced number of proliferative tumor cells. (Figure 5B).

### 3.8 ML and cisplatin have an additive interaction in H82 cell

Since we did not see a clear synergistic effect by adding ML to cisplatin in the mouse xenograft model, we tested for synergy in vitro in H82 cells. The expected drug combination responses were calculated based on the ZIP reference model.^18^ The model calculated a ZIP synergy score of 7.22 (*p* = 0.08) (Figure S5). This suggests that the interaction of ML and cisplatin is likely additive, with a trend for a weak synergistic effect that is not statistically significant.

## 4 DISCUSSION

Despite initial sensitivity to chemotherapy, SCLC is characterized by subsequent recurrence, metastasis, and resistance to treatment.^19^ While a targeted therapy approach has remained lacking, biological insights from the last decade into molecular subtypes may provide guidance in predicting response to certain therapeutic modalities. One important example of this paradigm is Myc expression as a biomarker for predicting treatment response to Aurora kinase inhibitors.^8^, ^20^

Myc influences expression of up to 15% of the human genome and functions to regulate multiple cancer‐relevant pathways including cell proliferation, differentiation, metabolism, and apoptosis.^21^ Even though C‐myc is implicated as a critical oncogene in a broad range of human cancers, research aiming at directly inhibiting C‐myc has yielded limited success. However, several indirect approaches to target C‐myc signaling are being investigated as potential anticancer treatments.^22^ Multiple strategies to target oncogenic myc proteins have been proposed, some of which are promising, but clinical applicability remains to be seen. Thus, additional study is needed to develop effective therapies that target myc‐driven cancers. Herein we introduce a novel approach in targeting myc in SCLC using the natural product mistletoe lectin.

We build on our prior findings in hepatocellular carcinoma, where treatment with mistletoe extract results in down‐regulation of C‐myc and slowing tumor growth.^14^ We apply this concept to SCLC and expand it to include another member of the myc family, N‐myc, that is also associated with worse prognosis^11^ and chemoresistance^9^ in SCLC. We demonstrate that ML treatment leads to down‐regulation of C‐myc and N‐myc protein expression in two SCLC cell lines, and to inhibition of cell growth. This change in myc protein expression is not due to decreased transcription of the respective myc genes, as demonstrated by the lack of drop in mRNA levels of C‐myc and N‐myc. On the contrary, mRNA levels have a trend to go up. We have previously shown that ML decreases myc protein levels by decreasing protein stability through regulating post‐translational modifications, specifically decreasing phosphorylation of S62 and slightly increasing phosphorylation of T58.^14^ This phosphorylation pattern promotes myc degradation.^23^ We suspect the uptrend in myc mRNA levels to be due to the negative autoregulation that myc has on its own transcription.^24^

Both H82 and H69 cells were much more sensitive to ML treatment than H196 cells. It is tempting to hypothesize that this is due to the lack of detectable levels of myc expression in H196 cells, but a larger sample of cell lines is needed to test this hypothesis. To get more direct evidence of myc mediating the effects of ML, we over‐expressed C‐myc and N‐myc in another SCLC cell line, SHP‐77. These cells have detectable C‐myc expression at baseline. In addition, we found them to be responsive to ML, with significantly increased sensitivity following over‐expression of either C‐myc or N‐myc. This suggests that the effect of ML on viability of SCLC cell lines is at least partially dependent on the expression of myc proteins.

In our mouse xenograft model, we found that ML treatment administered subcutaneously was tolerated and resulted in slowing of tumor growth compared to control treatment. The combination of ML and cisplatin did not seem to have a significant added effect on tumor size, although it did result in histologic evidence of increased apoptosis and decreased tumor cell proliferation compared to either treatment alone. These results are consistent with our finding of an additive interaction between ML and cisplatin in vitro but not a significant synergistic effect.

Mistletoe lectins exhibit anti‐cancer activity in multiple cancer types, which appears to partly by direct effect on cancer cells inducing apoptosis, and partly by an immunostimulatory effect.^25^ To our knowledge, we are the first to describe an application for ML in SCLC. Other studies that examined mistletoe in lung cancer were limited to non‐small‐cell lung cancer (NSLC). For instance, ML was found to inhibit the growth of two NSCLC cell lines in vitro by inducing apoptosis.^26^ In a phase II clinical trial in advanced NSCLC, the mistletoe preparation iscador was studied as a complimentary treatment to chemotherapy. It was not found to have an effect on quality of life or total adverse events but resulted in less frequent chemotherapy dose reductions, severe non‐hematological side effects, and hospitalizations.^27^

Our results showing that ML induces apoptosis in SCLC are consistent with several studies that demonstrated similar effects in different cancer types, including acute myeloid leukemia,^28^ Ewing's sarcoma,^29^ hepatocellular carcinoma^14^ and NSCLC.^26^ One of the strengths of our study is the use of both in vitro cell culture models and an in vivo mouse xenograft model to validate our results. However, our models lacked the capability to assess the immune effect, since our cell culture consisted of only the SCLC line and our mouse xenograft was in athymic mice. Future studies to examine the immune modulation effects of ML could utilize genetically engineered mouse models of SCLC that retain mouse immune function, or tumor xenografts in mice with “humanized” immune system. This is of interest because some of the anti‐tumor effects of mistletoe extracts have been attributed to immunomodulatory activity. For example, in advanced oral cancer, local injection of mistletoe extract resulted in stimulation of dendritic cells and activation of macrophage polarization followed by induced cytotoxicity.^30^ Clinical studies have demonstrated that mistletoe treatment induced increased granulocyte and eosinophil counts,^31^ lymphocytes and NK cells,^32^ and levels of several pro‐inflammatory cytokines.^33^

Future studies could utilize patient‐derived xenografts as a tool to study whether myc expression can serve as a biomarker predicting response to ML treatment. Additionally, with mistletoe extracts being available to administer to patients, a clinical trial studying safety and efficacy of a mistletoe preparation in patients with SCLC will likely be feasible in the foreseeable future.

In conclusion, we show that mistletoe lectin inhibits growth of myc‐amplified SCLC cell lines in vitro and in vivo and induces cancer cell apoptosis. This is the first report of an anti‐cancer effect of a mistletoe preparation in SCLC.

## AUTHOR CONTRIBUTIONS

**Afshin Dowlati:** Conceptualization (supporting); resources (supporting). **Betsy Gauthier:** Investigation (equal). **Eric Yuan:** Investigation (supporting). **Goutham Narla:** Conceptualization (supporting); writing – review and editing (equal). **Mohammad A Shatat:** Conceptualization (equal); funding acquisition (lead); investigation (equal); writing – original draft (lead). **Peiying Yang:** Conceptualization (supporting); writing – review and editing (equal). **Richard T Lee:** Conceptualization (equal); funding acquisition (supporting); resources (supporting); writing – review and editing (equal). **Suzy Yoon:** Investigation (supporting).

## FUNDING INFORMATION

This research was funded by the Department of Defense USAMRAA Award W81XWH1910646 (to MAS) and the Case Research Institute of the Case Western Reserve University and University Hospitals Cleveland Medical Center.

## CONFLICTS OF INTEREST

The authors have no relevant conflict of interest to declare.

## ETHICS APPROVAL

All animal experiments were approved by the IACUC at Case Western Reserve University.

## Supporting information


Appendix S1.

## Data Availability

The data that supports the findings of this study are available in the supplementary material of this article.
