# Supplementary material for: Mistletoe lectin inhibits growth of Myc‐amplified small‐cell lung cancer
Source: Cancer Med. 2022 Dec 23;12(7):8378–87. doi: 10.1002/cam4.5558 (PMC10134353; doi:10.1002/cam4.5558)
Supplement: Supplementary file 1 — Appendix S1. [file CAM4-12-8378-s001.docx]

**Mistletoe Lectin Inhibits Growth of Myc-amplified Small Cell Lung Cancer**

Supplemental Data

| **Table S1: Sequences of Primers used in QPCR** | | |
| --- | --- | --- |
| **Gene** | **Forward primer** | **Reverse primer** |
| MYC | CACCGAGTCGTAGTCGAGGT | TTTCGGGTAGTGGAAAACCA |
| MYCN | CACAGTGACCACGTCGATTT | TTCACAAGGCCCTCAGTACCTC |
| 18S ribosomal RNA | GCTTAATTTGACTCAACACGGA | AGCTATCAATCTGTCAATCCTGTC |
| Beta-Actin | CTCGCCTTTGCCGATCC | TCTCCATGTCGTCCCAGTTG |

| **Table S2: GI50 of ML in SCLC cell lines**. | |
| --- | --- |
| **Cell line** | **GI50 (ng/ml) (95% CI)** |
| H82 | 6.31 (5.35 to 7.46) |
| H69 | 6.23 (5.18 to 7.50) |
| H196 | 78.74 (65.43 to 95.31) |

| **Table S3: Fraction of H82 cells undergoing apoptosis for each treatment condition** | |
| --- | --- |
| **Treatment** | **% cells undergoing apoptosis** |
| Control | 4.30 |
| ML 8ng/ml (24h) | 7.33 |
| ML 16ng/ml (24h) | 9.07 |
| ML 8ng/ml (48h) | 7.94 |
| ML 16ng/ml (48h) | 22.9 |

| **Table S4: Effect of myc over-expression on GI50 in SHP77 cells** | |
| --- | --- |
| **Cell line** | **ML GI50 (ng/ml) (95% CI)** |
| SHP77-hcRed | 12.29 (10.20-14.88) |
| SHP77-C-Myc | 6.50 (5.47-7.71) |
| SHP77-GFP | 7.90 (6.78-9.20) |
| SHP77-N-myc | 3.44 (2.82-4.16) |


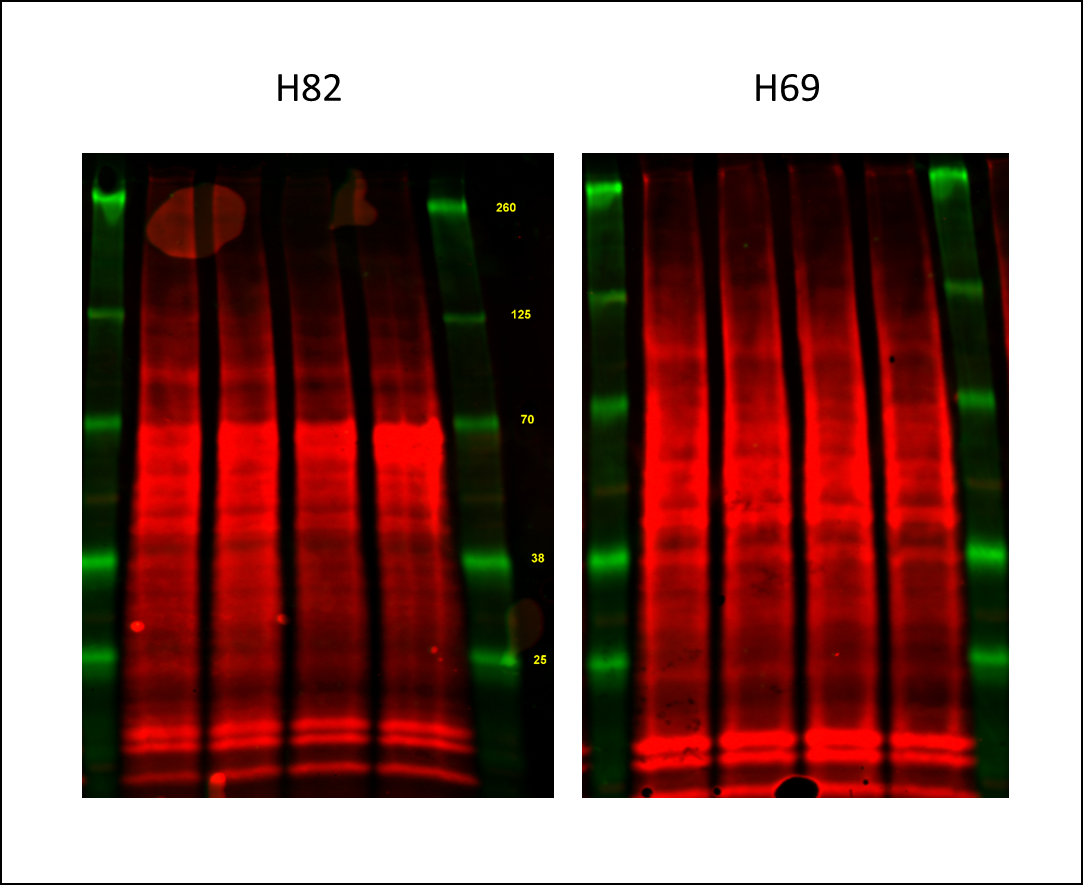

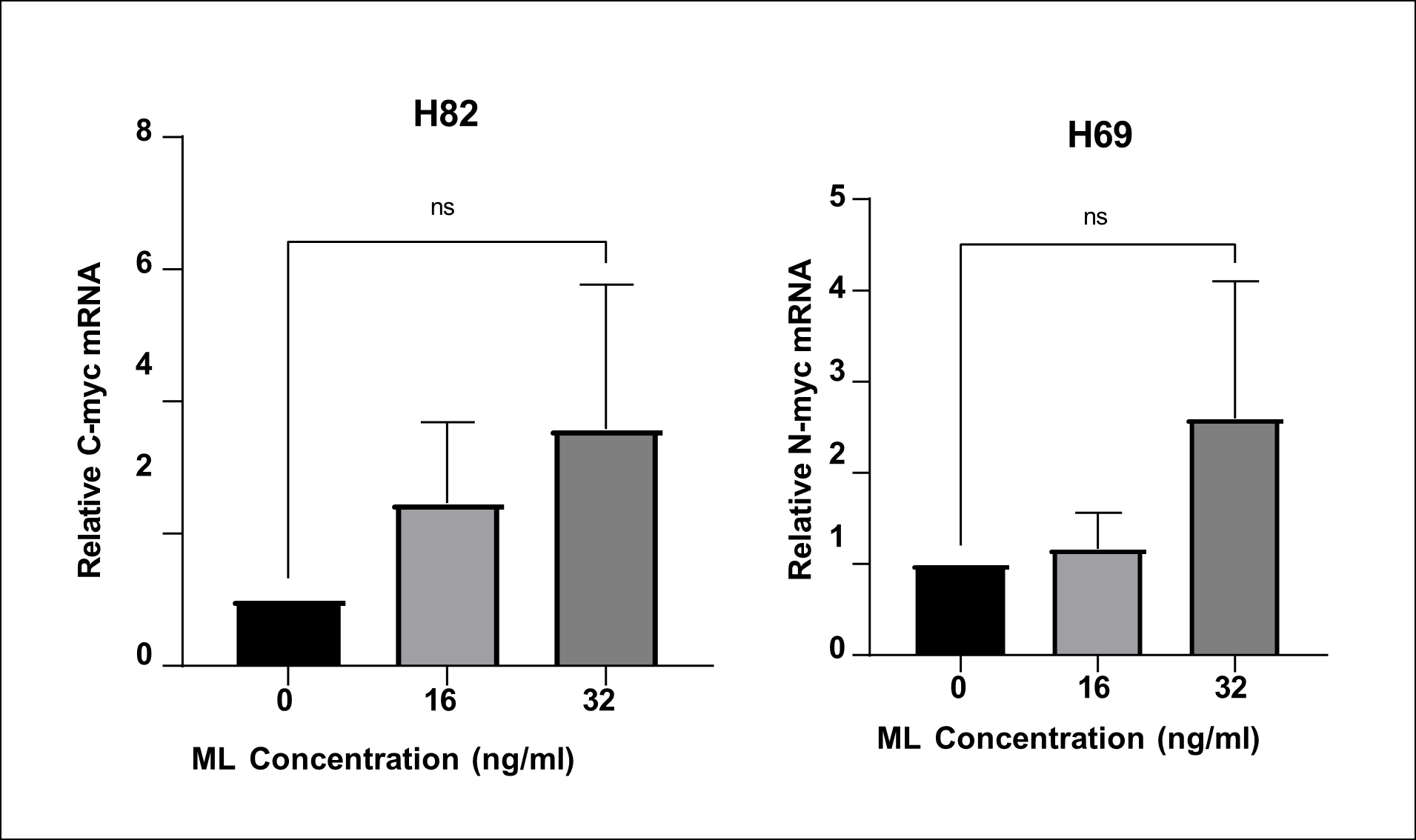

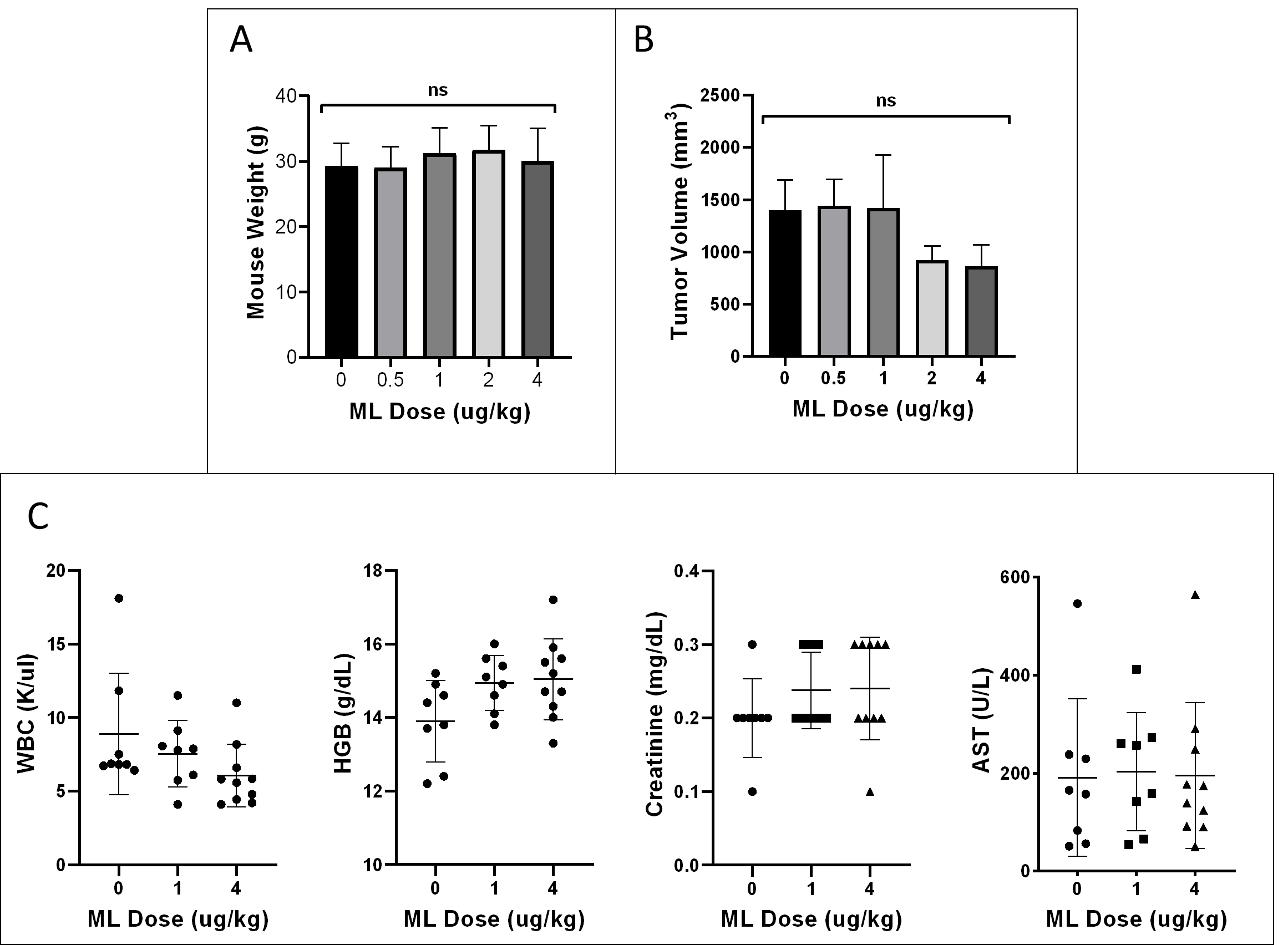


**Figure S1**: Total protein loading for the immunoblots shown in figure 2A visualized using Revert™ 700 stain.

**Figure S2**: RT-QPCR results of MYC mRNA in H82 cells and MYCN in H69 following incubation with the indicated concentrations of ML for 24 hours. Results were from three independent experiments and were not statistically significant.

**Figure S3:** Pilot Studies of ML Treatment in Mice Assessing Potential Toxicity and effect on tumor size.

1. Increasing doses of ML were administered subcutaneously to 8-week-old athymic nude mice every 3 days for 4 weeks. Mouse weight on day 28 is shown.
2. H82 cells (4x10^6^/mouse) were injected into the right flank of athymic nude mice to form tumor xenografts. Once tumor volume reached an average of 200mm^3^, treatment with increasing doses of ML 3 times per week was initiated for a total of 4 doses. Tumor volume is shown after the fourth dose.
3. Blood was collected at the end of the experiment in A and a subset of blood samples were analyzed for complete blood count and chemistry. Results are shown for white blood cell count (WBC), hemoglobin (HGB), creatinine and aspartate aminotransferase (AST). None of the results showed a statistically significant difference between the groups.


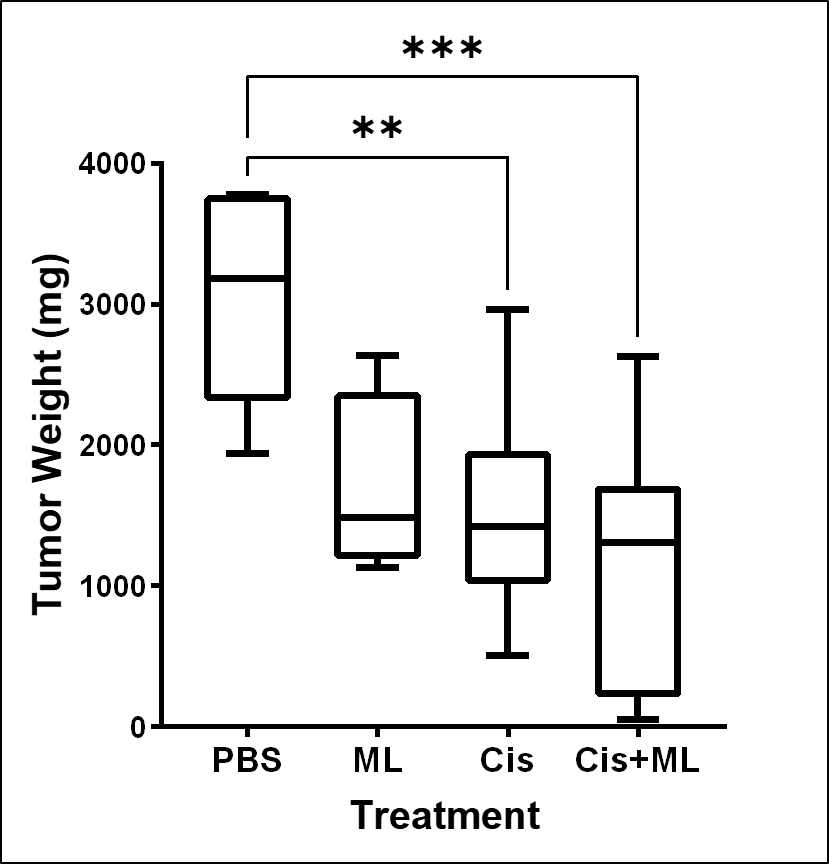


**Figure S4**: Weight of excised tumors at the end of the experiment.


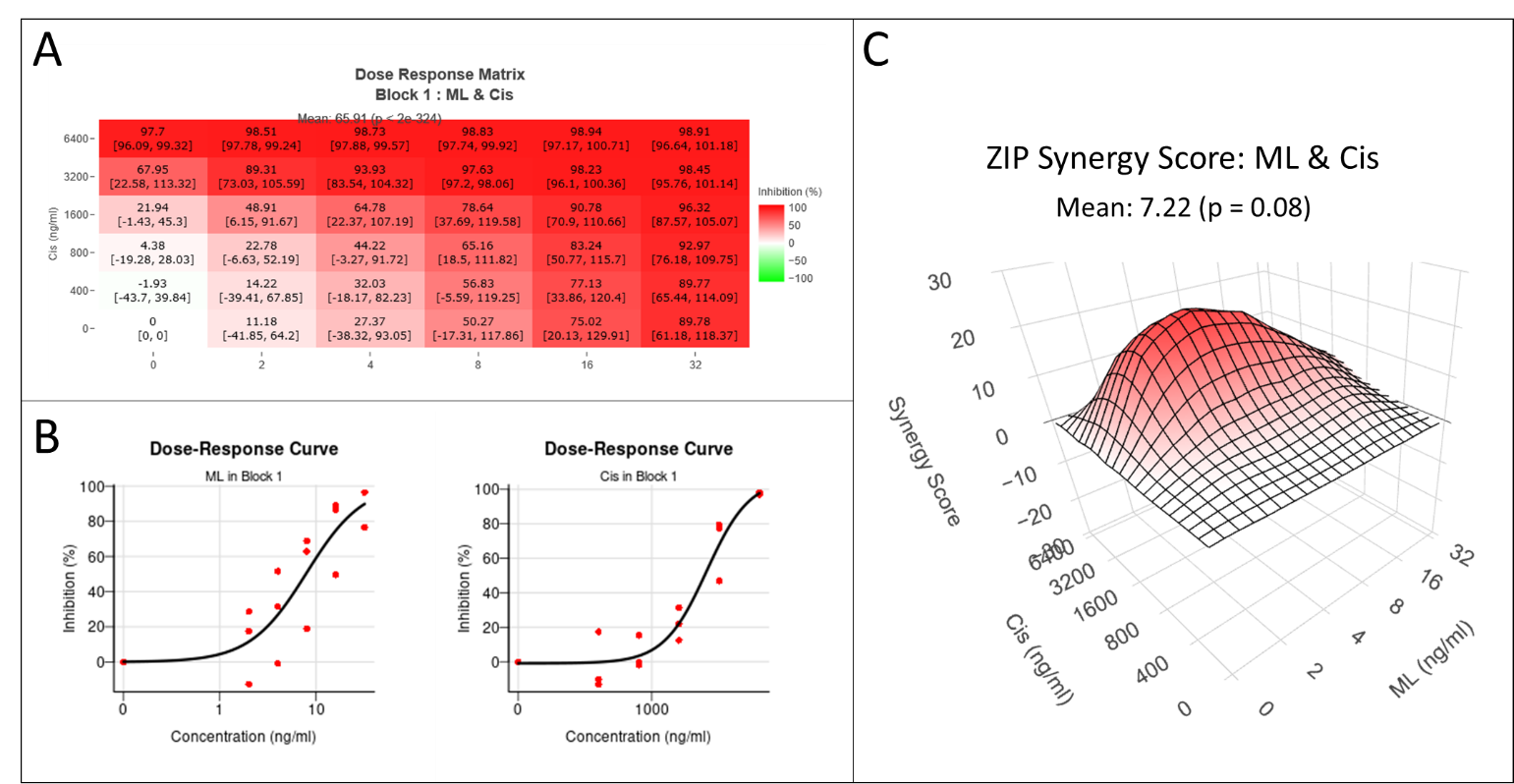


**Figure S5**: Effect of combination treatment with ML and cisplatin in H82 cells. Results were obtained by uploading cell viability data from three independent experiments into the SynergyFinder web application.

1. Dose response matrix of different combination of ML and cisplatin expressed as percent growth inhibition with 95% CI.
2. Individual dose response curves for ML and cisplatin.
3. 3D plot of the interaction between ML and cisplatin using ZIP reference model.
